# The untapped potential of cell culture in disentangling insect-microbial relationships

**DOI:** 10.20517/mrr.2023.66

**Published:** 2024-02-29

**Authors:** Christine V. Macpherson, Brendan A. Daisley, Elizabeth Mallory, Emma Allen-Vercoe

**Affiliations:** Department of Molecular and Cellular Biology, University of Guelph, Guelph N1G 2W1, ON, Canada.

**Keywords:** Cell culture, host-microbe interactions, insects, gut microbiome

## Abstract

Cell culture is a powerful technique for the investigation of molecular mechanisms fundamental to health and disease in a diverse array of organisms. Cell lines offer several advantages, namely their simplistic approach and high degree of reproducibility. One field where cell culture has proven particularly useful is the study of the microbiome, where cell culture has led to the illumination of microbial influences on host immunity, nutrition, and physiology. Thus far, researchers have focused cell culture work predominantly on humans, but the growing field of insect microbiome research stands to benefit greatly from its application. Insects constitute one of Earth’s most diverse and ancient life forms and, just as with humans, possess microbiomes with great significance to their health. Insects, which play critical roles in supporting food security and ecological stability, are facing increasing threats from agricultural intensification, climate change, and pesticide use. As the microbiome is closely tied to host health, gaining a more robust understanding is of increasing importance. In this review, we assert that the cultivation and utilization of insect gut cell lines in microbiome research will bridge critical knowledge gaps essential for informing insect management practices in a world under pressure.

## INTRODUCTION

Cell culture typically refers to the growth and maintenance of eukaryotic cells outside of their native environment under controlled laboratory conditions^[1]^. Cultured cells may be derived from humans, insects, other animals, plants, and fungi. By breaking down the organism into discrete parts, cell culture models offer a high-resolution view of the current subject, free of *in vivo* confounders^[1]^. As well, the homogeneity of cell culture, with a singular cell type and a well-defined genetic profile, allows for exceptional reproducibility^[1]^. The application of cell culture has revolutionized *in vitro* research endeavors, especially in genetics, pathology, and microbiology.

*In vivo* animal models are a commonly used approach to scientific research, serving as valuable tools for confirming *in vitro* findings and investigating various biological processes, diseases, and potential treatments. These models allow researchers to study complex physiological and pathological phenomena in a controlled environment. Although insect animal models raise less ethical concerns and debate than vertebrate models, there are several limitations when completing *in vivo* research. Practical barriers, encompassing the acquisition and maintenance of these insects, can prove daunting for researchers. Such issues include obtaining administrative approval, securing sufficient space and equipment for rearing insects, or possessing the knowledge to supply the environmental conditions and nutritional needs required. Further, in studies that investigate microbe-host tissue interactions, researchers face the intricate task of mitigating confounding factors linked to the host. This involves addressing challenges like immunological response and the potential off-target effects of other microbes within the host. Creating germ-free insect models, a crucial step to limit these confounding factors, is labour-intensive and may induce detrimental changes to the form and function of the host. Another notable concern is the overwintering of insects in temperate or continental climates, which can impact research timelines and reproducibility. Recognizing these challenges, researchers are exploring alternative models, such as cell lines, for preliminary and mechanistic studies within controlled environments, offering a potential workaround to some of the obstacles inherent in using live insect models.

One field where cell culture models have proven to be uniquely powerful is in the study of the microbiome, which is the community of microorganisms (and their genetic potential, metabolites, structural elements, and environmental conditions) living within the intestinal tract of the host^[2]^. Cell culture of human epithelial cells, particularly of intestinal origin, has improved our understanding of how microbes (or microbial metabolites) impact intestinal transepithelial permeability^[3]^, immune signaling^[4]^, and cellular proliferation mechanisms relevant to cancer^[5]^.

Despite great promise, researchers have not yet adopted this approach concerning the insect microbiome. Similar to the human gut microbiome, the insect gut microbiome serves as a critical intermediary between the environment and host health, aiding in digestion, detoxification, and combating pathogens^[6,7]^, yet it remains poorly understood. Crucial to both ecosystem health and global food security, insects are facing rising threats of habitat destruction, pesticide use, and antimicrobials^[8,9]^. Given their importance and vulnerability, it is essential to understand the microbes so inextricably tied to their health. In order to study host-gut microbiome interactions in insects, tissue-specific intestinal cell lines must be obtained from insect hosts. Overall, cell lines can be separated into two major categories: primary and immortalized. Primary cell lines are obtained directly from insects and maintained under stringent laboratory conditions for short periods of time before they eventually die out. Immortalized lines are an asset because they allow for laboratory manipulation without the need for available live insect sources. Of the 1,500 immortalized insect cell lines openly available, a mere 0.012% represent midgut tissue^[10]^. Of this fraction, only 9 of the over 1 million known insect species are represented^[10,11]^. In this review, we explore the characteristics of insect cell lines, their use in studying host-microbial interactions, and emerging techniques for the immortalization of novel insect gut cell lines.

## A BRIEF HISTORY OF INSECT CELL CULTURE

The roots of insect research at the cellular level can be traced back to 1912, when Glaser and Chapman examined how wilt disease affected spongy moth (*Lymantria dispar*) hemocytes - key immune effector cells of invertebrates that participate in phagocytosis, encapsulation, and clotting systems^[12]^. In the following decades, insect cell culture techniques continued to advance, resulting in the successful establishment of multiple primary cell lines^[13]^. Continuous cell lines, however, remained elusive during this early work, as cell lines were unable to survive beyond 3 months^[13]^. This milestone is generally accepted to have been finally achieved by Grace (1962) when he derived a continuous cell line from the ovarian tissue of emperor gum moth (*Opodipthera eucalypti*) pupae^[14]^. Since this time, insect cell culture has blossomed along with advancements in aseptic techniques and complex media formulations. A notable cell line, Sf-21, which was produced by Vaughn *et al*. in 1977, is used in the mass production of baculovirus stocks and recombinant proteins for human vaccine production to date^[15,16]^. Insect cells have also been used in infectious disease research, pesticide screening, and “bio-bots” (small robots incorporating animal biomass), and have the potential to be used as a food source^[13,17,18]^. At present, over 1,500 insect cell lines spanning 7 orders of taxonomy are cataloged on Cellosaurus^[11]^, displaying significant progress in the field over 80 years.

## THE GROWTH REQUIREMENTS OF INSECT CELL LINES

**Figure 1:**
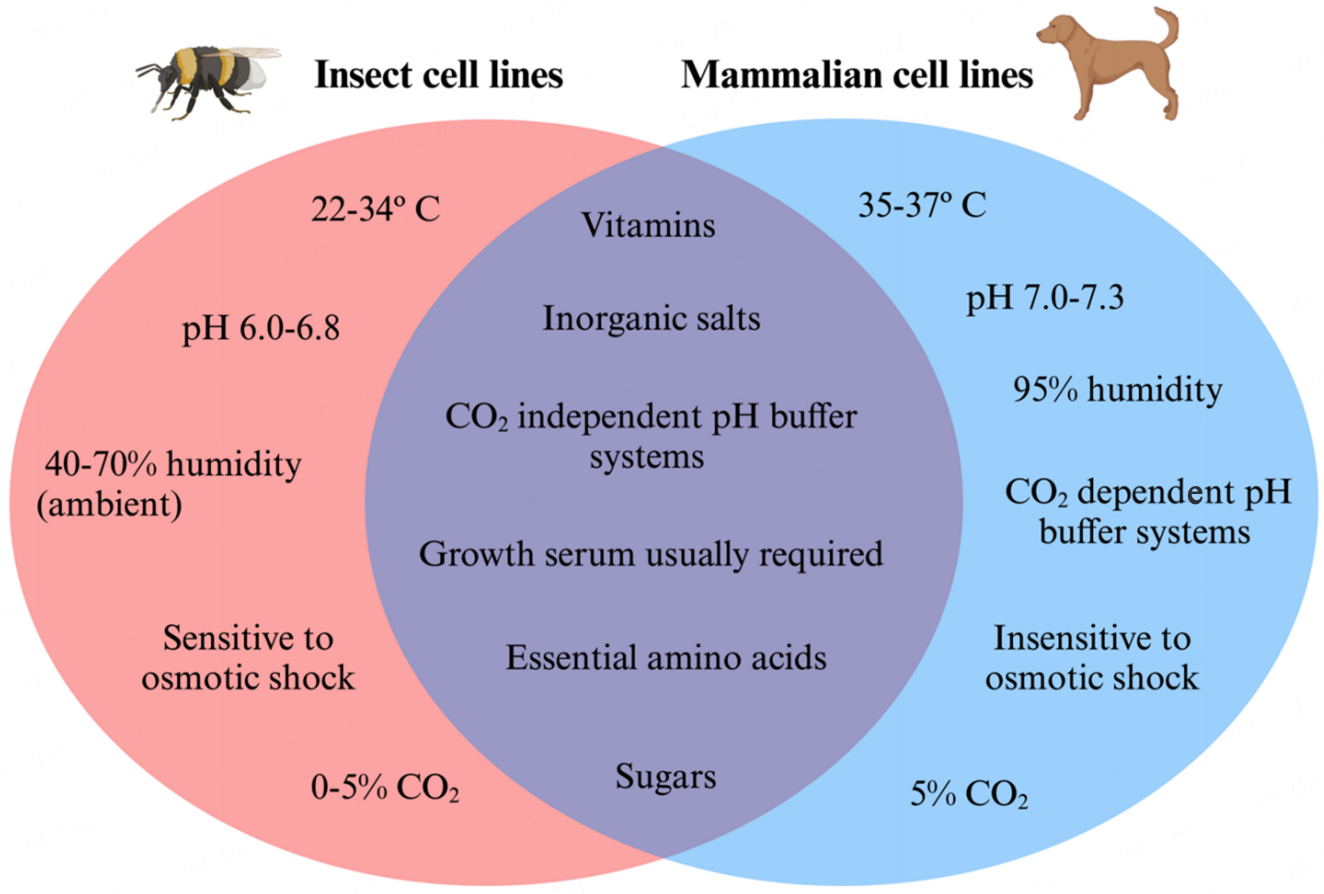
Venn diagram illustrating culture condition requirements of insect and mammalian cell lines. Insect and mammalian cell lines share many nutritional requirements, but differ in their environmental conditions, with insect cell lines notably requiring a lower resource demand. Created with BioRender.com^[20]^.

Insect cell lines exhibit broad adaptability to culture conditions and many lines can be successfully cultivated in temperatures ranging from 22-34 °C, 0%-5% CO_2_, pH 6.0-6.8, and ambient humidity^[15,19]^. In contrast, mammalian cells typically require tight adherence to *in vivo* conditions during incubation, including a consistent temperature of 35-37 °C, 5% CO_2_, pH 7.0-7.3, and 95% relative humidity [Figure 1]^[15]^. Thus, insect cell lines are less fastidious compared to mammalian lines, most notably lacking the requirement of a CO_2_ incubator. Insect cells also grow more rapidly, are less sensitive to changes in pH, and express significantly more protein than mammalian cells^[15]^. This versatility may be, at least in part, due to the exothermic nature of insects which requires them to tolerate a range of ambient temperatures.

Several types of media have been developed that satisfy the nutritional requirements and chemical conditions needed by insect cells^[15,21]^. In general, insect cells require essential amino acids, inorganic salts, sugars, and vitamins^[21]^. Additionally, media is often supplemented with growth-promoting substances, namely insect hemolymph or fetal bovine serum^[21]^. These conditions are similar to mammalian cells and are subject to modification depending on the cell line.

The control of pH in insect cell lines is primarily achieved with a phosphate buffer system, which does not require a CO_2_ incubator to function^[15]^. Mammalian cells, by contrast, commonly utilize either HEPES [4-(2-hydroxyethyl)-1-piperazineethanesulfonic acid], a CO_2_-independent buffer system, or a bicarbonate buffer, which relies on regulated CO_2_ conditions to effectively control pH^[22]^. HEPES is not usually recommended for insect cell culture; however, some literature has reported its use, namely in AmE-711, a spontaneous cell line derived from embryonic western honey bee (*Apis mellifera*) tissue^[15,19]^. Insect cell lines display a markedly elevated rate of oxygen consumption and a decreased rate of lactate production in comparison to mammalian cells. This discrepancy may be exaggerated in cell culture, as a large proportion of mammalian cell lines are derived from cancerous tissue. A well-documented phenomenon, the Warburg effect, is the observation that cancerous tissue exhibits reduced oxygen consumption and heightened lactate production compared to healthy tissue^[23,24]^. Nonetheless, insect cells’ increased gas exchange (and therefore CO_2_ accumulation) necessitates the introduction of base to increase pH^[15]^. The addition of base increases the osmolarity of the media and, in turn, increases the risk of osmotic shock in cell culture^[15]^. The degree to which low lactate production ameliorates the acidic effect of heightened CO_2_ production in insect cells is unknown and could be a promising area to explore in future studies.

## ZOOMING IN: THE POTENTIAL OF INSECT GUT CELL LINES TO IMPROVE OUR UNDERSTANDING OF THE MICROBIOME

Cell lines have served as valuable tools in the understanding of host-microbe interactions in insects. The predominant focus of these studies, however, has been the investigation of host-pathogen relationships; nominal research has explored the impact of symbionts on the host and no research has studied microbial communities, including those of the gut. In this review, we separate the study of pathogens from the study of the microbiome and emphasize studies using cell lines to understand host-symbiont relationships and the impact of microbial communities on insect health. Studies have yet to explore the use of insect gut cell lines to understand host-microbial relationships, although several studies utilize other tissues for this purpose.

One study that utilized insect cell culture to investigate a symbiont-host interaction in mosquitos (*Aedes fluviatilis*) observed that cultured embryonic cells infected with the obligate endosymbiont *Wolbachia pipientis* displayed improved energy performance and innate immune system activation^[25]^. Other studies have employed cell lines to elucidate the molecular mechanisms of bacterial infiltration into host cells. For example, a study found that an unclassified *Rickettsia* sp., an endosymbiont of rice green leafhopper (*Nephotettix cincticeps*) sperm, could migrate to the nucleus of two larval cell lines belonging to different phylogenetic orders^[26]^. This work provides unique insight into endosymbiotic theory and the vertical transmission of microbes between hosts. Another avenue for the use of insect cell culture in microbiome research is in the culture of sensitive microorganisms that benefit from the presence of host tissue^[27]^. These cultured microbes may then be used to perform *in vitro* or *in vivo* assays. Several intracellular microbes, such as the previously mentioned *W. pipientis*, have been cultured in this manner^[28]^. This diverse array of uses underscores both the versatility and potential of insect cell lines in microbiome research.

The insect gut microbiome has evolved alongside its host over millions of years, forming an intimate and often life-sustaining bond^[29,30]^. Microbes provide insect hosts with essential nutrient provisioning, aid in digestion, and confer resistance to pathogens^[6,7]^. Illustrating this, bacterial strains isolated from the gut of the bark beetle (*Dendroctonus rhizophagus*) have been shown to hydrolyze pectin, cellulose, xylan, starch, lipids, and esters - thereby allowing the host organism to derive nutrition from otherwise indigestible substrates^[31]^. By increasing metabolic capacity and diversity, the insect microbiome thus allows the exploitation of a wider variety of food sources. Further, bacterial metabolites isolated from the gut of the fruit fly (*Drosophila melanogaster*) have been shown to increase immune gene expression of pathways that promote the activity of Relish/NF-κB - a family of pleiotropic transcription factors that are highly conserved across Animalia^[32,33]^. As innate immunity is greatly conserved across flies and mammals, understanding the microbial modulation of these pathways in insects can also enrich our comprehension of immune signaling in diverse animal systems. Altogether, the simple growth requirements and efficiency of insect cell lines, as well as the applicability of many conserved cellular systems across insects and vertebrates, position insect cell culture as a powerful model system for studying host-microbe interactions relevant to a very wide range of host species.

Despite their applicability, a significant constraint in the use of insect cell lines for microbiome research is the limited availability of continuous cell lines derived from relevant insect gut tissue. Gut cell lines have been established for only nine insect species, and many of the most heavily studied insects, such as *A. mellifera* and *D. melanogaster*, are not represented^[10]^. This paucity of gut-relevant cell lines restricts the range of host-microbe dynamics that can be studied *in vitro*, while also limiting the study of tissue-specific interactions. Cell lines derived from embryos or non-gut tissue, for example, may not capture the full complexity of *in vivo* gut environments, including physical, chemical, and microbial interactions.

An additional factor to consider when investigating the microbiome using cell lines is how closely a cell line approximates the gut environment. Primary cell lines, derived from isolated tissue, retain most of their *in vivo* functionality and can include gut ultrastructure, but only survive for short periods in cell culture^[34]^. When considering immortalized cell lines, very little has been characterized in detail, whether derived from humans or insects^[35]^. The Caco-2 cell line derived from human tissue, which will be discussed in detail in this review, is one of the only cell lines characterized enough to determine a relative approximation of gut tissue conditions (i.e., polarized monolayer, forms tight junctions, expresses many receptors and enzymes associated with gut tissues)^[36,37]^.

Recently, insect cell lines derived from various tissues have been subjected to detailed genome or transcriptome profiling to gain insights into characteristics important for recombinant protein expression^[38-40]^. However, when considering insect gut cell lines, genotypic and phenotypic characterization is lacking.

The RP-HzGUT-AW1 cell line, derived from Lepidoptera member *Helicoverpa zea*, is one of the first insect cell lines to be characterized via transcriptomics for expression of insect intestinal epithelial cell gene and intestinal stem cell markers and, therefore, is the most characterized in gut approximation^[41]^. RP-HzGUT-AW1 exhibits some, but not all, gene markers approximated for intestinal stem cell markers and differentiated epithelial cells, suggesting the cell line consists of progenitor cells, which form smooth muscle junctions between cells^[41]^. A mechanism to differentiate the progenitor cells further was not discussed^[41]^. Going forward, initial characterization of gut cell lines should be performed at the transcriptomic level so that the troubleshooting and development of other gut features, such as recreating structural organization and peritrophic membrane formation, can be attempted. Advanced bio-mimetic technologies such as organ-on-chip and organoid models have yet to be adapted to insect tissues, although they could be explored to address these issues.

The lack of basic insect gut cell line characterization is a major hurdle in the development of biologically relevant cell lines that respond to stimuli (e.g., microbial metabolites, chemical treatments of interest) in a manner that is predictable and comparable to *in vivo* conditions. Thus, while current insect cell cultures offer unique insights, their limited representation of genuine, characterized gut tissues can constrain the depth and overall breadth of host-microbe interaction studies. Here, we highlight lessons learned from human gut-derived cell lines and describe several of the latest techniques that show promise for enabling targeted derivation of insect gut cell lines.

### Caco-2 and the microbiome: an exemplar approach

Cell culture techniques have been effectively leveraged to gain insight into mammalian epithelial cell-microbial relationships over the past several decades. The human cell line model of the gut epithelium most widely used for this purpose is likely the Caco-2 line, derived by Fogh in 1977 from a human colon adenocarcinoma biopsy. Despite their derivatization from the colon, these cells behave most similarly to small intestine epithelial tissue, both biochemically and morphologically^[42]^. Demonstrating their broad-spectrum application, Caco-2 cells have been used in thousands of studies to illuminate the pathogenesis of deleterious microbes, probiotic formulations, and immune-microbe interactions^[43-45]^. For example, Caco-2 cells have been employed to confirm both the invasiveness of, and the cytokine response to *Fusobacterium nucleatum* - a bacterium associated with colorectal cancer - in a relevant intestinal cell model^[45,46]^. Additionally, this cell line has been used to evaluate the potential beneficial properties of the yeast *Kluyveromyces marxianus,* strain B0299^[44]^, where it was observed that the yeast dampened the release of pro-inflammatory cytokines by cells treated with lipopolysaccharide, an inflammatory stimulus, which ultimately led to the yeast being considered as a therapeutically useful probiotic^[44]^.

A further aspect that extends the applicability of the Caco-2 cell model to biomedical science is that the cells can be co-cultured with other cell types and incorporated into several advanced biomimetic models. For instance, the co-culture of Caco-2 and Raji B cells, a lymphoblast-like cell line, facilitates the production of a model of specialized microfold epithelial cells, which overlay the gut-associated lymphoid tissues^[47]^. This model can subsequently be utilized to study the transport of microbial-derived micro- and nano-compounds across this epithelial barrier cell type^[47,48]^. Caco-2 cells have also been integrated into organ-on-chip models, which are micro-biomimetic systems that incorporate multiple cell types and provide a framework for cellular growth^[49]^. This model system more closely represents *in vivo* tissue, improving the efficacy of *in vitro* studies.

Considering the extensive insights gained from using mammalian cell lines such as Caco-2, similar methodologies should be applied to insect models. However, since spontaneously immortalized tissue is rare to identify in insects under natural circumstances, the pivotal challenge of deriving gut-specific cell types lies primarily in the isolation procedure rather than the general maintenance steps. The tailored methodologies that show the most promise for isolating gut-derived cell lines able to grow continuously (i.e., which exhibit immortalization) under laboratory conditions are discussed below.

## PRODUCING IMMORTALIZED INSECT GUT CELL LINES

### Cell types relevant to insect gut cell line development

**Figure 2:**
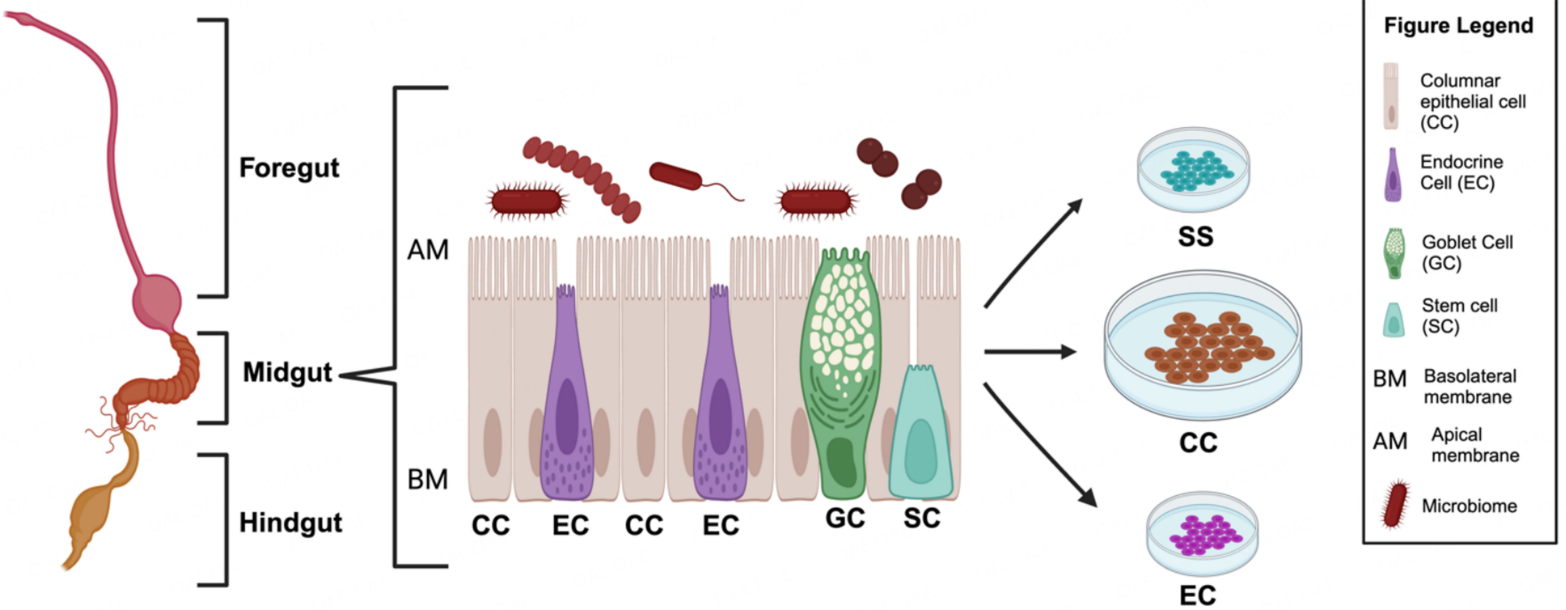
Targeted zones to produce insect gut cell lines. Schematic representation of the insect digestion system, which can be broken down into three regions: the foregut, midgut, and hindgut. The midgut is the primary choice from which to derive cell lines on account of it playing a key role in shaping the gut microbiome and being the principal location of nutrient absorption, pathogen defense, and the production of signaling molecules^[50]^. The midgut has several cell types, including columnar epithelial cells (CC), endocrine cells (EC), goblet cells (GC), and stem cells (SC)^[50]^. Only the most relevant of the top five cell types are shown. Columnar epithelial cells are the most common, followed by endocrine cells and stem cells, making these cells the first selection for cell culture^[50]^. Created with BioRender.com^[20]^.

Contrasting the human gut epithelium, the insect gut epithelium is relatively simple, consisting of two major cell types: columnar epithelial cells and endocrine cells [Figure 2]^[50]^. Additionally, stem cells, goblet cells, and copper cells (responsible for acidification of the digestive tract) are often present [Figure 2]^[50,51]^. Columnar epithelial cells closely resemble the stereotypical enterocytes found in humans and function similarly. We suggest that columnar epithelial cells, as the most prevalent cell type in the insect gut epithelium, represent a good target for successful cultivation during cell line isolation procedures. It is important to note that non-target cell types, such as muscle cells, exist within the insect gut as well^[50]^. As a result, cell lines are not always composed of their target cell type and should be verified by, at minimum, microscopy prior to further experimentation.

### Methods to attain immortalized insect gut cell lines

Cell lines are considered immortalized once they no longer possess the ability to respond to cell cycle controls. These controls, present at the G_1_ and G_2_ cell cycle checkpoints, limit cellular proliferation and, when required, lead to cellular senescence or apoptosis^[52]^. Bypassing these controls can, on rare occasions, occur naturally in a process known as spontaneous immortalization^[50]^. Alternatively, this can be achieved deliberately, for example, through the insertion of proto-oncogenes, pathogen invasion, Clustered Regularly Interspaced Short Palindromic Repeats (CRISPR)-Cas9, and exposure to carcinogenic chemicals or ionizing radiation^[53-56]^. Immortalization is particularly difficult to achieve in mature tissue, such as the gut epithelium, because of its limited replicative potential and greater likelihood of senescence^[48]^. Notwithstanding these difficulties, many insect cell lines have been successfully produced from mature tissue. Table 1 lists the currently available insect cell lines that have been immortalized. Several common techniques for the production of insect gut cell lines are relevant to discuss.

**Table 1:** Categorization of the 17 available immortalized insect gut cell lines

**Cell line name(s)**	**Species**	**Age at isolation**	**Suggested cell type**	**Date** **published**	**Cellosaurus accession #(s)**
BCIRL-HZ-MG8	Corn earworm moth (*Helicoverpa zea*)	Unspecified	Unspecified (untested)	2003^[57]^	CVCL_ZF01
BPH22	Painted grasshopper (*Poekilocerus pictus*)	Nymph	Stem cells (differentiates into goblet and epithelial cells)	2010^[58]^	CVCL_A2PR
BTI-Tn-MG1, MG1-ht33, MG1-ht35	Cabbage looper (*Trichoplusia ni*)	Fifth instar larva	Unspecified, mixed morphology	1991^[59]^	CVCL_Z093, CVCL_UF21, CVCL_UF22
CT/BCIRL-SfMG-0617-KZ, CT/BCIRL-SfMG1-0611-E7-KZ, CT/BCIRL-SfMG1-0611-E8-KZ, CT/BCIRL-SfMG1-0611-KZ	Fall armyworm (*Spodoptera frugiperda*)	Fifth instar larval stage, sixth instar larva	Unspecified, mixed morphology (muscle and epithelial origin, has residual epithelial cells)	2019^[60]^	CVCL_UZ18, CVCL_C2T6, CVCL_C2T7, CVCL_UZ19
FPMI-CF-200, FPMI-CF-203, FPMI-CF-204, FPMI-CF-205, IPRI-CF-1	Spruce budworm moth (*Choristoneura fumiferana*)	Adult, neonate larva	Unspecified, mixed morphology	1993^[61]^	CVCL_WV83, CVCL_Z469, CVCL_WV85, CVCL_WV84, CVCL_Z473
HNU-Ha-MG1	Cotton bollworm (*Helicoverpa armigera*)	Fourth instar larva	Unspecified, mixed morphology	2015^[62]^	CVCL_HF52
LSTM-AG-55	African malaria mosquito (*Anopheles gambiae*)	First instar larva	Epithelial-type cells	1972^[63]^	CVCL_Z360
RPW-1	Red palm weevil (*Rhynchophorus ferrugineus*)	Fifth instar larva	Midgut epithelial cells (untested)	2013^[64]^	CVCL_A2PS

Cell line characteristics were gathered from the *Cellosaurus* cell line catalog^[10]^. All cell lines were produced through spontaneous immortalization.

### Spontaneous immortalization

Spontaneous immortalization occurs when a cell line is immortalized without exposure to carcinogens or direct genetic manipulation^[50]^. Continuous passaging of a primary cell line increases the likelihood of random epigenetic changes or chromosomal rearrangements^[50]^. Often, a mutation occurs in the p53 pathway^[50,65]^. Although considered a rare event, spontaneous immortalization has led to the production of all currently existing insect gut cell lines^[10]^. Notably, midgut cell lines have been successfully produced in this manner, including CT/BCIRL-SfMG1-0611-E8-KZ (Fall armyworm midgut)^[10]^.

### Malignant neoplasms

Neoplasia has been reported in various insect tissues, including hemolymph, epithelial tissue, and neural tissue^[66-68]^. Many human cell lines, including Caco-2, have been derived from a malignant neoplasm, but no insect gut cell lines have been established using this approach. This is particularly notable, as *D. melanogaster* is a model organism for the study of colon cancer due to its tractability and ease of genetic manipulation^[66,69]^. Samples of insect tumors could represent an untapped goldmine of undiscovered cell lines.

### Insertion of proto-oncogenes

Proto-oncogenes play essential roles in the normal growth and development of cells, however, they can potentially create malignant neoplasms if excessive copies accumulate or mutations occur, creating an oncogene^[70]^. Proto-oncogenes can be introduced to cells through a method known as lipofection, which transfects DNA using a synthetic cationic lipid complex^[71,72]^. Lipofection of the human *C-myc* proto-oncogene into embryonic *A. mellifera* cells successfully produced the MYN9 cell line^[54]^. Considered a master regulator, excessive *C-myc* can lead to immortalization by inciting reactive oxygen species production and promoting p53 phosphorylation, disrupting normal cell cycle function^[71,73]^. Another proto-oncogene used in cell line production is *ras*, an important regulator of proliferation, growth, and survival^[71]^. Both the Ras[V12]-H1 and Ras[V12]-H7 *D. melanogaster* cell lines include mutations in the *ras* proto-oncogene^[74]^.

### Entomopathogens

Certain pathogens incite tumorigenesis or reduce apoptosis in the gut epithelial tissue of insects. In one study, the European spruce sawfly (*Gilpinia hercyniae*) developed multiple midgut tumors after exposure to a polyhedral virus^[75]^. Additionally, *Nosema cerenae*, a fungal pathogen of *A. mellifera*, reduces apoptosis of midgut epithelial cells by increasing the transcription of the *inhibitor of apoptosis-2* gene^[76]^. The direct application of entomopathogens in insect cell line production has yet to be explored; however, this is not to suggest that pathogens may not have already played a critical role. Both existing *A. mellifera* cell lines (AmE-711 and MYN9) are known to be infected with deformed wing virus^[77]^, which could have contributed to their immortalization.

### CRISPR-Cas9

CRISPR-Cas9 allows for the targeted insertion and deletion of genes from a host genome. CRISPR-Cas9 technology has recently been used to immortalize mature human prostate epithelial cells of the PrEC cell line^[56]^. In this cell line, the inactivation of *CDKN2A* at two loci induced immortalization, but also maintained the characteristics of normal cells, including a normal p53 response^[56]^. CRISPR-Cas9 has also been used to edit several existing insect cell lines, including insect cell lines Sf-21 and S2R+^[78,79]^. Despite great potential, CRISPR-Cas9 has not yet been employed to produce a novel insect cell line. Future research endeavors may benefit from editing genes that have been empirically linked to midgut tumorigenesis in *D. melanogaster*, a commonly used insect model of colon cancer^[66,69]^. Two of these genes are *APC* and *ras*, which control the activation of the Wnt signaling pathway and drive the proliferation and migration of cells, respectively^[66]^. These two genes could be key targets in future studies.

### Radiation

Ionizing radiation (X-rays and Gamma-rays) induces direct DNA damage in the form of single- and double-stranded breaks and indirect DNA damage through the production of reactive oxygen species^[80]^. Following the theory of radiation hormesis, low-dose radiation is purported to improve cellular health, but high-dose radiation is extremely damaging and results in DNA breaks^[81]^. Radiation causes apoptosis in the majority of exposed cells, but has the potential to produce providential mutations, leading to immortalization in a subset of these cells. The KMST-6 human fibroblast cell line was produced in this way using Gamma radiation^[53]^, illustrating the promise of this technique. Furthermore, literature from as early as 1928 has associated X-ray exposure with genetic mutation in insects, indicating promise for future work using radiation to deliberately transform insect cells to immortalize them^[69]^.

## INSECT GUT CELL LINES HAVE THE POTENTIAL TO ADDRESS MICROBIOME-RELATED KNOWLEDGE GAPS

### Defining a “healthy” insect microbiome

A substantial knowledge gap that could be addressed by insect gut cell lines is the balance of microbes that constitutes a healthy, or “eubiotic,” gut microbiome^[82]^. Eubiosis in insects is poorly defined, as is the opposite state, dysbiosis. This, in large part, is due to the context-dependent nature and inherent subjectivity of the gut microbiome^[83]^. Additionally, very few insect species have well-studied microbiomes, furthering this lack of understanding.

One theory that attempts to define microbiome health in insects follows the Anna Karenina principle of “Happy families are all alike; every unhappy family is unhappy in its own way”^[84,85]^. According to this framework, once a eubiotic species balance is achieved, any deviation from this species balance will destabilize the system, leading to dysbiosis. This principle applies well to social insect species, which possess highly conserved and vertically transmitted microbiome compositions^[86,87]^. However, the theory fails to consider solitary insect microbiomes, which more often mimic the composition of their environment^[6,88]^. Other researchers have argued that the level of connectedness determines the health of the microbiome, rather than the balance of species^[84]^. Just as for the human microbiome, it is unknown what truly constitutes a “healthy” insect microbiome, especially when the correct balance differs from species to species. Insect gut cell lines hold the potential to elucidate the roles of microbes and their contributions towards eubiosis or dysbiosis within the insect gut, and the animal as a whole.

### Culturing the uncultured

For most animals, the gut houses the greatest number of microorganisms in the body, many of which remain uncultured^[72,89]^, meaning culture cannot be achieved using traditional microbiological methods, likely because agars and broths utilized in laboratory settings do not replicate the complex physiological and chemical conditions of the gut environment^[29]^. One option to improve culture conditions is the employment of bioreactor models, which show great promise in cultivating entire gut-derived communities, but still lack the host cell component^[90]^. In future studies, bioreactors could be linked to cell culture through microbial metabolites. Cell-free supernatant from bioreactors is a source of microbial metabolites, which can be filter-sterilized and applied to insect cell lines to replicate host-microbial metabolite interactions. Since much of the interaction between a microbe and an animal cell is mediated through metabolite signaling, the use of metabolites alone helps preserve cell line integrity while understanding host-microbe interactions. This may be achieved by assessing cytokine release, localization within a host cell, and the up- or downregulation of host gene expression.

In addition, since cell culture replicates the gut tissue microenvironment, it allows for the cultivation and isolation of sensitive microbes that are strictly dependent on the presence of host cells^[29]^. For example, obligate intracellular microbes, which rely entirely on eukaryotic host cells, must be cultured in this manner^[29]^, including the endosymbiont *W. pipientis,* which was first cultured and isolated utilizing the Aa23 cell line, derived from the Asian tiger mosquito (*Aedes albopictus*)^[28]^. Through the use of insect gut cell lines, previously uncultured bacteria, archaea, and fungi can be isolated, enhancing our comprehension of insect pathogens, commensals, and symbionts. Further, undiscovered constituents of the insect microbiome may possess characteristics valuable to human industry and pharmaceuticals.

In highlighting the efficacy of cell lines for investigating facultative symbionts, the 2018 study by Chevignon *et al*. stands out as a notable example^[91]^. To explore the “unculturable” aphid symbiont *Hamiltonella defensa*, crucial for safeguarding its host against parasitoid wasps, Chevignon *et al*. leveraged the immortalized TN5 insect cell line^[91]^. The utilization of cell lines in cultivating *H. defensa* yielded a higher abundance of the symbiont compared to *in vivo* cultures^[91]^. This enhancement enabled the researchers to successfully generate the complete genomes for various *H. defensa* strains, thereby unraveling the genetic variance of strains and genome elements responsible for the protective abilities exhibited by *H. defensa*^[91]^.

### Understanding the microbiome’s toxin defense systems

The insect microbiome serves as a critical barrier between toxins and host tissue. The scientific literature has documented the ability of insect gut microbes to mitigate toxin exposure to the host through the breakdown of external compounds, such as insecticides, as well as host-secreted wastes^[6]^. The insect microbiome also exhibits a capacity to adapt to toxin exposure. Namely, microbes of the brown plant hopper (*Nilaparvata lugens*), an agricultural pest, displayed heightened expression of detoxifying genes, including *NlCYP6ER1*, after repeated exposure to insecticides^[92]^. Additionally, a commensal bacterial species of *D. melanogaster*, *Lacticaseibacillus* (formerly *Lactobacillus*) *rhamnosus*, reduced the absorption of organophosphate insecticides by the host^[93]^. Furthermore, several lactic acid bacteria strains, isolated from the gut of managed *A. mellifera*, had the ability to detoxify representative insecticides in cell line models^[94]^. While encouraging, it is important to note that none of the cell lines used (ovarian insect Sf-9, rat intestinal IEC-6, and human intestinal Caco-2) were of *A. mellifera* origin and the detoxification abilities observed were contingent on the cell lines employed^[94]^. The relevance of cross-species studies is thus context-dependent, underscoring the importance of deriving and employing species-specific cell lines in insect-related research to ensure the accuracy of results.

Despite progress in the field, a significant knowledge gap remains regarding the impact of specific toxins on host tissue in isolation as well as in the presence of various resident and foreign microbes. This gap may be addressed by insect gut cell lines, which in turn could help to advance pest management strategies, and to shield vulnerable species from the noxious effects of toxin exposure.

### Exploring specific symbiont-derived pathogen defense in host environment

The application of cell lines in the investigation of host-pathogen-symbiont interactions will promote a more comprehensive understanding of symbiont-derived defense and its impact on the host, while avoiding confounders associated with host models. These confounders include the inherent colonization of the host by other microbiome members, which could make any interaction studies non-specific, or would require the use of germ-free hosts (which often are not available). Few studies have used cell line models to look at such interactions. One study of note used a rotating wall vessel-derived (RWV) 3-D HT29 organotype model, composed of a human adenocarcinoma cell line with epithelial morphology (HT-29), to investigate the interactions between symbiont, human host, and pathogen during infection^[95]^. The RWV model was coinfected with commensal bacteria *Lactobacillus reuteri* and the pathogen *Salmonella enterica*, in order to determine the protective ability of *L. reuteri* and its impact on the host^[95]^. The study revealed that *L. reuteri* reduced pathogen growth and adhesion to HT-29 cells, leading to *L. reuteri* being considered a potential candidate for probiotic therapy against *Salmonella* infection^[95]^. This study highlights the potential of cell culture to elucidate mechanisms underlying symbiont-derived pathogen defense, as well as the repercussions for all cells involved.

A wider range of host-specific gut cell lines could improve insect work by creating an accurate environment to inoculate both pathogen and potential symbiont, resulting in more natural responses to what would happen within the specific host. With the use of insect gut cell lines, research of this nature could help to identify safe probiotic candidate bacteria against the most relevant “incurable” insect pathogens, such as *Paenibacillus larvae* in honeybees, gain insights on microbiome members important for host defense, and the impact of both pathogen and symbiont presence in host tissues^[96]^.

## LIMITATIONS

The key limitation associated with cell culture is its inability to fully recapitulate *in vivo* organ and tissue environments^[1]^. While the simplistic model of cell culture is efficacious in the study of isolated variables, it consequently cannot assess the impact of the same variables on complex systems, such as the reproductive and immune systems. This discrepancy could be addressed in the future through the production of co-culture and microfluidic systems. Another limitation of cell lines is that they cannot reproduce the dramatic gut remodeling that many insects undergo between life stages^[50]^. Additionally, immortalized cells tend to behave differently than the primary cells from which they were derived. This is due to changes in important regulatory genes necessary to prevent senescence. One notable difference between the currently available immortalized insect midgut cells and primary cells of the same origin is the lack of polarization, which limits studies on epithelial barrier function^[97]^. As a result of these limitations, it is important to understand cell culture as a tool for preliminary and mechanistic work, which must be validated with appropriate *in vivo* studies.

## CONCLUSION

Insect gut cell lines offer a high-resolution and relevant model for the exploration of the microbiome and its complex interactions with host nutrition, immunity, and toxicology. Despite decades of research in the fields of the insect microbiome and insect cell culture, limited integration has occurred between the two. This starkly contrasts the study of the human microbiome, which has utilized cell culture extensively. The time has come to explore novel ways to study host-microbiome relationships in insects in the laboratory, and cell lines represent an important tool to exploit. This is becoming more important as insects continue to face unrelenting threats as a result of human development and habitat encroachment.

## DECLARATIONS

### Copyright

© The Author(s) 2024.

## References

[1] Segeritz C-P, Vallier L

[2] Berg G, Rybakova D, Fischer D (2020). Microbiome definition re-visited: old concepts and new challenges. Microbiome.

[3] Lopez-Escalera S, Wellejus A (2022). Evaluation of Caco-2 and human intestinal epithelial cells as *in vitro* models of colonic and small intestinal integrity. Biochem Biophys Rep.

[4] (2005). van Nuenen MHMC, de Ligt RAF, Doornbos RP, van der Woude JCJ, Kuipers EJ, Venema K. The influence of microbial metabolites on human intestinal epithelial cells and macrophages in vitro. FEMS Immunol Med Microbiol.

[5] Jahani-Sherafat S, Azimirad M, Ghasemian-Safaei H (2019). The effect of intestinal microbiota metabolites on HT29 cell line using MTT method in patients with colorectal cancer. Gastroenterol Hepatol Bed Bench.

[6] Engel P, Moran NA (2013). The gut microbiota of insects - diversity in structure and function. FEMS Microbiol Rev.

[7] Jing TZ, Qi FH, Wang ZY (2020). Most dominant roles of insect gut bacteria: digestion, detoxification, or essential nutrient provision?. Microbiome.

[8] Goulson D (2019). The insect apocalypse, and why it matters. Curr Biol.

[9] Janicki J, Dickie G, Scarr S, Chowdhury J https://www.reuters.com/graphics/GLOBAL-ENVIRONMENT/INSECT-APOCALYPSE/egpbykdxjvq/.

[10] Cellosaurus - a knowledge resource on cell lines https://www.cellosaurus.org/.

[11] Stork NE (2018). How many species of insects and other terrestrial arthropods are there on earth?. Annu Rev Entomol.

[12] Glaser RW, Chapman JW (1912). Studies on the wilt disease, or “flacheria” of the gypsy moth. Science.

[13] Smagghe G, Goodman CL, Stanley D (2009). Insect cell culture and applications to research and pest management. In Vitro Cell Dev Biol Anim.

[14] Grace TDC (1962). Establishment of four strains of cells from insect tissues grown *in vitro*. Nature.

[15] Drugmand JC https://dial.uclouvain.be/pr/boreal/en/object/boreal%3A4586.

[16] Vaughn JL, Goodwin RH, Tompkins GJ, McCawley P (1977). The establishment of two cell lines from the insect *Spodoptera frugiperda* (lepidoptera; noctuidae). In Vitro.

[17] Rubio NR, Fish KD, Trimmer BA, Kaplan DL (2019). Possibilities for engineered insect tissue as a food source. Front Sustain Food Syst.

[18] Gisder S, Möckel N, Linde A, Genersch E (2011). A cell culture model for *Nosema ceranae* and *Nosema apis* allows new insights into the life cycle of these important honey bee-pathogenic microsporidia. Environ Microbiol.

[19] Goblirsch MJ, Spivak MS, Kurtti TJ (2013). A cell line resource derived from honey bee (*Apis mellifera*) embryonic tissues. PLoS One.

[20] https://www.biorender.com/.

[21] Mitsuhashi J

[22] Michl J, Park KC, Swietach P (2019). Evidence-based guidelines for controlling pH in mammalian live-cell culture systems. Commun Biol.

[23] Singh L, Nair L, Kumar D (2023). Hypoxia induced lactate acidosis modulates tumor microenvironment and lipid reprogramming to sustain the cancer cell survival. Front Oncol.

[24] Warburg O (1925). The metabolism of carcinoma cells. J Cancer Res.

[25] Conceição CC, da Silva JN, Arcanjo A (2021). *Aedes fluviatilis* cell lines as new tools to study metabolic and immune interactions in mosquito-Wolbachia symbiosis. Sci Rep.

[26] Watanabe K, Yukuhiro F, Matsuura Y, Fukatsu T, Noda H (2014). Intrasperm vertical symbiont transmission. Proc Natl Acad Sci U S A.

[27] Brandt JW, Chevignon G, Oliver KM, Strand MR (2017). Culture of an aphid heritable symbiont demonstrates its direct role in defence against parasitoids. Proc Biol Sci.

[28] O’Neill SL, Pettigrew MM, Sinkins SP, Braig HR, Andreadis TG, Tesh RB (1997). *In vitro* cultivation of *Wolbachia pipientis* in an *Aedes albopictus* cell line. Insect Mol Biol.

[29] Rajagopal R (2009). Beneficial interactions between insects and gut bacteria. Indian J Microbiol.

[30] Menezes C, Vollet-Neto A, Marsaioli AJ (2015). A Brazilian social bee must cultivate fungus to survive. Curr Biol.

[31] Briones-Roblero CI, Rodríguez-Díaz R, Santiago-Cruz JA, Zúñiga G, Rivera-Orduña FN (2017). Degradation capacities of bacteria and yeasts isolated from the gut of *Dendroctonus rhizophagus* (Curculionidae: Scolytinae). Folia Microbiol.

[32] Gilmore TD (1999). The Rel/NF-κB signal transduction pathway: introduction. Oncogene.

[33] Buchon N, Silverman N, Cherry S (2014). Immunity in *Drosophila melanogaster* - from microbial recognition to whole-organism physiology. Nat Rev Immunol.

[34] Marian B (2002). In vitro models for the identification and characterization of tumor-promoting and protective factors for colon carcinogenesis. Food Chem Toxicol.

[35] Langerholc T, Maragkoudakis PA, Wollgast J, Gradisnik L, Cencic A (2011). Novel and established intestinal cell line models - an indispensable tool in food science and nutrition. Trends Food Sci Technol.

[36] Costa J, Ahluwalia A (2019). Advances and current challenges in intestinal *in vitro* model engineering: a digest. Front Bioeng Biotechnol.

[37] Cencic A, Langerholc T (2010). Functional cell models of the gut and their applications in food microbiology - a review. Int J Food Microbiol.

[38] Koczka K, Peters P, Ernst W, Himmelbauer H, Nika L, Grabherr R (2018). Comparative transcriptome analysis of a *Trichoplusia ni* cell line reveals distinct host responses to intracellular and secreted protein products expressed by recombinant baculoviruses. J Biotechnol.

[39] Fu Y, Yang Y, Zhang H (2018). The genome of the Hi5 germ cell line from *Trichoplusia ni*, an agricultural pest and novel model for small RNA biology. Elife.

[40] Wei L, Cao L, Miao Y (2017). Transcriptome analysis of *Spodoptera frugiperda* 9 (Sf9) cells infected with baculovirus, AcMNPV or AcMNPV-*Bm*K IT. Biotechnol Lett.

[41] Vorgia E, Lamprousi M, Denecke S (2021). Functional characterization and transcriptomic profiling of a spheroid-forming midgut cell line from *Helicoverpa zea* (Lepidoptera: Noctuidae). Insect Biochem Mol Biol.

[42] Sun H, Chow ECY, Liu S, Du Y, Pang KS (2008). The Caco-2 cell monolayer: usefulness and limitations. Expert Opin Drug Metab Toxicol.

[43] Bahrami B, Child MW, Macfarlane S, Macfarlane GT (2011). Adherence and cytokine induction in Caco-2 cells by bacterial populations from a three-stage continuous-culture model of the large intestine. Appl Environ Microbiol.

[44] Maccaferri S, Klinder A, Brigidi P, Cavina P, Costabile A (2012). Potential probiotic *Kluyveromyces marxianus* B0399 modulates the immune response in Caco-2 cells and peripheral blood mononuclear cells and impacts the human gut microbiota in an *in vitro* colonic model system. Appl Environ Microbiol.

[45] Castellarin M, Warren RL, Freeman JD (2012). *Fusobacterium nucleatum* infection is prevalent in human colorectal carcinoma. Genome Res.

[46] Dharmani P, Strauss J, Ambrose C, Allen-Vercoe E, Chadee K (2011). *Fusobacterium nucleatum* infection of colonic cells stimulates MUC2 mucin and tumor necrosis factor alpha. Infect Immun.

[47] Kleiveland CR

[48] Lozoya-Agullo I, Araújo F, González-Álvarez I (2017). Usefulness of Caco-2/HT29-MTX and Caco-2/HT29-MTX/Raji B coculture models to predict intestinal and colonic permeability compared to Caco-2 monoculture. Mol Pharm.

[49] Singh D, Mathur A, Arora S, Roy S, Mahindroo N (2022). Journey of organ on a chip technology and its role in future healthcare scenario. Appl Surf Sci Adv.

[50] Caccia S, Casartelli M, Tettamanti G (2019). The amazing complexity of insect midgut cells: types, peculiarities, and functions. Cell Tissue Res.

[51] Dubreuil RR (2004). Copper cells and stomach acid secretion in the *Drosophila* midgut. Int J Biochem Cell Biol.

[52] Wright WE, Shay JW (1992). The two-stage mechanism controlling cellular senescence and immortalization. Exp Gerontol.

[53] Namba M, Nishitani K, Hyodoh F, Fukushima F, Kimoto T (1985). Neoplastic transformation of human diploid fibroblasts (KMST-6) by treatment with ^60^Co gamma rays. Int J Cancer.

[54] Kitagishi Y, Okumura N, Yoshida H, Nishimura Y, Takahashi J, Matsuda S (2011). Long-term cultivation of in vitro *Apis mellifera* cells by gene transfer of human c-myc proto-oncogene. In Vitro Cell Dev Biol Anim.

[55] Bryan TM, Reddel RR (1994). SV40-induced immortalization of human cells. Crit Rev Oncog.

[56] Zhao Z, Fowle H, Valentine H (2021). Immortalization of human primary prostate epithelial cells via CRISPR inactivation of the *CDKN2A* locus and expression of telomerase. Prostate Cancer Prostatic Dis.

[57] Pringle FM, Johnson KN, Goodman CL, McIntosh AH, Ball LA (2003). Providence virus: a new member of the *tetraviridae* that infects cultured insect cells. Virology.

[58] Kharat KR, Sawant MV, Peter S, Hardikar BP (2010). Development and characterization of new cell line BPH22 from midgut epithelial cells of *Poekilocerus pictus* (Fabricius, 1775). In Vitro Cell Dev Biol Anim.

[59] Granados RR https://patents.google.com/patent/US5298418A/en.

[60] Zhou K, Goodman CL, Ringbauer J Jr, Song Q, Beerntsen B, Stanley D (2020). Establishment of two midgut cell lines from the fall armyworm, *Spodoptera frugiperda* (Lepidoptera: Noctuidae). In Vitro Cell Dev Biol Anim.

[62] Li J, He F, Yang Y (2015). Establishment and characterization of a novel cell line from midgut tissue of *Helicoverpa* armigera (Lepidoptera: Noctuidae). In Vitro Cell Dev Biol Anim.

[63] Marhoul Z, Pudney M (1972). A mosquito cell line (mos. 55) from *Anopheles gambiae* larva. Trans R Soc Trop Med Hyg.

[64] Aljabr AM, Rizwan-ul-Haq M, Hussain A, Al-Mubarak AI, Al-Ayied HY (2014). Establishing midgut cell culture from *Rhynchophorus ferrugineus* (Olivier) and toxicity assessment against ten different insecticides. In Vitro Cell Dev Biol Anim.

[65] Harvey DM, Levine AJ (1991). p53 alteration is a common event in the spontaneous immortalization of primary BALB/c murine embryo fibroblasts. Genes Dev.

[66] Martorell Ò, Merlos-Suárez A, Campbell K (2014). Conserved mechanisms of tumorigenesis in the *Drosophila* adult midgut. PLoS One.

[67] Pastor-Pareja JC, Wu M, Xu T (2008). An innate immune response of blood cells to tumors and tissue damage in Drosophila. Dis Model Mech.

[68] Scharrer B (1953). Insect tumors induced by nerve severance: incidence and mortality. Cancer Res.

[69] Villegas SN (2019). One hundred years of *Drosophila* cancer research: no longer in solitude. Dis Model Mech.

[70] https://www.nature.com/scitable/topicpage/proto-oncogenes-to-oncogenes-to-cancer-883/.

[71] Frühauf MI, Barcelos LDS, Botton NY (2021). Alternatives for obtaining a continuous cell line from *Apis mellifera*. Cienc Rural.

[72] Felgner PL, Gadek TR, Holm M (1987). Lipofection: a highly efficient, lipid-mediated DNA-transfection procedure. Proc Natl Acad Sci U S A.

[73] Poole CJ, van Riggelen J (2017). MYC - master regulator of the cancer epigenome and transcriptome. Genes.

[74] Dequéant ML, Fagegaltier D, Hu Y (2015). Discovery of progenitor cell signatures by time-series synexpression analysis during *Drosophila* embryonic cell immortalization. Proc Natl Acad Sci U S A.

[75] BIRD FT (1949). Tumours associated with a virus infection in an insect. Nature.

[76] Kurze C, Le Conte Y, Dussaubat C (2015). *Nosema* tolerant honeybees (*Apis mellifera*) escape parasitic manipulation of apoptosis. PLoS One.

[77] https://entomology.ca.uky.edu/aginsectcellsdatabase?combine=&field_cell_order_tid=303&field_cell_species_tid=All&field_cell_stage_tid=All&field_cell_status_tid=All&field_cell_tissue_tid=All.

[78] Sari-Ak D, Alomari O, Shomali RA, Lim J, Thimiri Govinda Raj DB (2022). Advances in CRISPR-Cas9 for the baculovirus vector system: a systematic review. Viruses.

[79] Nweke EE, Thimiri Govinda Raj DB (2021). Chapter one - development of insect cell line using CRISPR technology. In: Singh V, editor. Progress in molecular biology and translational science. Prog Mol Biol Transl Sci.

[80] Carlos-Reyes A, Muñiz-Lino MA, Romero-Garcia S, López-Camarillo C, Hernández-de la Cruz ON (2021). Biological adaptations of tumor cells to radiation therapy. Front Oncol.

[81] Vaiserman A, Cuttler JM, Socol Y (2021). Low-dose ionizing radiation as a hormetin: experimental observations and therapeutic perspective for age-related disorders. Biogerontology.

[82] Iebba V, Totino V, Gagliardi A (2016). Eubiosis and dysbiosis: the two sides of the microbiota. New Microbiol.

[83] Daisley BA, Koenig D, Engelbrecht K (2021). Emerging connections between gut microbiome bioenergetics and chronic metabolic diseases. Cell Rep.

[84] Marasco R, Fusi M, Callegari M (2022). Destabilization of the bacterial interactome identifies nutrient restriction-induced dysbiosis in insect guts. Microbiol Spectr.

[85] Tolstoy L

[86] Sabree ZL, Huang CY, Arakawa G (2012). Genome shrinkage and loss of nutrient-providing potential in the obligate symbiont of the primitive termite *Mastotermes darwiniensis*. Appl Environ Microbiol.

[87] Su Q, Wang Q, Mu X (2021). Strain-level analysis reveals the vertical microbial transmission during the life cycle of bumblebee. Microbiome.

[88] De Landa GF, Alberoni D, Baffoni L (2023). The gut microbiome of solitary bees is mainly affected by pathogen assemblage and partially by land use. Environ Microbiome.

[89] Sender R, Fuchs S, Milo R (2016). Revised estimates for the number of human and bacteria cells in the body. PLoS Biol.

[90] Gianetto-Hill CM, Vancuren SJ, Daisley B (2023). The Robogut: a bioreactor model of the human colon for evaluation of gut microbial community ecology and function. Curr Protoc.

[91] Chevignon G, Boyd BM, Brandt JW, Oliver KM, Strand MR (2018). Culture-facilitated comparative genomics of the facultative symbiont *Hamiltonella defensa*. Genome Biol Evol.

[92] Zhang Y, Cai T, Yuan M (2023). Microbiome variation correlates with the insecticide susceptibility in different geographic strains of a significant agricultural pest, *Nilaparvata lugens*. NPJ Biofilms Microbiomes.

[93] Trinder M, McDowell TW, Daisley BA (2016). Probiotic *Lactobacillus rhamnosus* reduces organophosphate pesticide absorption and toxicity to *Drosophila melanogaster*. Appl Environ Microbiol.

[94] Leska A, Nowak A, Miśkiewicz K, Rosicka-Kaczmarek J (2022). Binding and detoxification of insecticides by potentially probiotic lactic acid bacteria isolated from honeybee (*Apis mellifera L*.) environment - an in vitro study. Cells.

[95] De Weirdt R, Crabbé A, Roos S (2012). Glycerol supplementation enhances *L. reuteri*’s protective effect against *S*. typhimurium colonization in a 3-D model of colonic epithelium. PLoS One.

[96] Daisley BA, Pitek AP, Mallory E (2023). Disentangling the microbial ecological factors impacting honey bee susceptibility to *Paenibacillus larvae* infection. Trends Microbiol.

[97] Swevers L, Denecke S, Vogelsang K, Geibel S, Vontas J (2021). Can the mammalian organoid technology be applied to the insect gut?. Pest Manag Sci.

